# RNA-Binding Proteins Play an Important Role in the Prognosis of Patients With Testicular Germ Cell Tumor

**DOI:** 10.3389/fgene.2021.610291

**Published:** 2021-03-11

**Authors:** Liangyu Yao, Rong Cong, Chengjian Ji, Xiang Zhou, Jiaochen Luan, Xianghu Meng, Ninghong Song

**Affiliations:** ^1^Department of Urology, The First Affiliated Hospital of Nanjing Medical University, Nanjing, China; ^2^Department of Urology, The Affiliated Kizilsu Kirghiz Autonomous Prefecture People’s Hospital of Nanjing Medical University, Artux, China

**Keywords:** testicular germ cell tumors, RNA-binding proteins, risk score, prognosis, nomogram

## Abstract

Testicular germ cell tumors (TGCTs) are common urological neoplasms in young adult males. The outcome of TGCT depends on pathologic type and tumor stage. RNA-binding proteins (RBPs) influence numerous cancers via post-transcriptional regulation. The prognostic importance of RBPs in TGCT has not been fully investigated. In this study, we set up a prognostic risk model of TGCT using six significantly differentially expressed RBPs, namely, *TRMT61A, POLR2J, DIS3L2, IFIH1, IGHMBP2*, and *NPM2*. The expression profiles were downloaded from The Cancer Genome Atlas (TCGA) and Genotype-Tissue Expression datasets. We observed by performing least absolute shrinkage and selection operator (LASSO) regression analyses that in the training cohort, the expression of six RBPs was correlated with disease-free survival in patients with TGCT. We assessed the specificity and sensitivity of 1-, 3-, 5-, and 10-year survival status prediction using receiver operating characteristic curve analysis and successfully validated using the test cohorts, the entire TCGA cohort, and Gene Expression Omnibus (GEO) datasets. Gene Ontology, Kyoto Encyclopedia of Genes and Genomes, and gene set enrichment analyses were carried out to seek the possible signaling pathways related with risk score. We also examined the association between the model based on six RBPs and different clinical characteristics. A nomogram was established for TGCT recurrence prediction. Consensus clustering analysis was carried out to identify the clusters of TGCT with different clinical outcomes. Ultimately, external validations of the six-gene risk score were performed by using the GSE3218 and GSE10783 datasets downloaded from the GEO database. In general, our study constructed a prognostic model based on six RBPs, which could serve as independent risk factor in TGCT, especially in seminoma, and might have brilliant clinical application value.

## Introduction

Testicular germ cell tumors (TGCTs) are the most common malignant neoplasms in young adult males between 20 and 40 years old despite being regarded as rare tumor types that account for only 1% of solid neoplasms in men (Tsili et al., 2019). TGCT has two main subtypes: seminoma and non-seminoma. The outcome depends on its pathologic type and tumor stage (Zhang et al., 2018). Although TGCT mortality is lower than 10% and the cure rate has reached 95% because of the application of cisplatin-based chemotherapy (Diamantopoulos and Kortsaris, 2010; Cheng et al., 2018), 10–15% of the patients are refractory to first-line chemotherapy and TGCT has poor outcome (Oechsle et al., 2011). Serum tumor markers, such as alpha fetoprotein (AFP), human chorionic gonadotropin (hCG), and lactose dehydrogenase (LDH), are widely applied clinically and effective in the diagnosis, staging, risk stratification, treatment, and evaluation of patients’ response to chemotherapy in TGCT (Marshall et al., 2019). Several studies also attempted to look for biomarkers related with prognosis of TGCT. MGMT and CALCA promoter methylation were found in connection with poor outcome in patients with TGCT (Martinelli et al., 2017). β-catenin was linked to suppressed immune infiltration and poor clinical features in TGCT (Chovanec et al., 2018). However, a risk model for TGCT prognosis prediction has not been designed so far.

RNA-binding proteins (RBPs) are identified as conserved proteins in eukaryotes that play critical roles in co-transcriptional and post-transcriptional gene regulation, particularly RNA maturation, turnover, localization, and translation (Glisovic et al., 2008). The dysregulation of RBPs in diverse cancers influences the expression and function of tumor-related proteins through mechanisms, such as post-transcriptional regulations, RBP–RNA networks, and results in cancer development (Pereira et al., 2017). The musashi RNA binding protein 2 stabilizes androgen receptor mRNA by targeting its 3′-untranslated region and drives prostate cancer progression (Zhao et al., 2020). Similarly, KHDRBS1 functions as a post-transcriptional regulator by binding to its target mRNAs and suppresses colon cancer metastasis (Yu et al., 2020). Notably, RBPs might be involved in spermatogenesis and TGCT. DAZL knockout in mouse germ cells leads to complete male sterility through the gradual loss of spermatogonial stem cells (SSCs), meiotic arrest, and spermatid arrest (Li et al., 2019). KHDRBS1 downregulation inhibits the proliferation, accelerates the apoptosis of germ cell, and thus causes spermatogenic defects in human testes (Li et al., 2014). The increased expression of LINC00162 in the nucleus could promote the proliferation of testicular embryonal carcinoma cells via miR-320a and miR-383 by binding to nucleolin, a kind of RBP (Lü et al., 2015). However, few researchers had focused on the prognostic value of RBPs in TGCT; therefore, our work is necessary.

In the current study, we explored the prognostic importance of RBPs in TGCT by analyzing the expression data of differentially expressed RBPs in TGCT obtained from online databases. The risk model constructed by Cox regression analyses and Lasso regression showed that expression of six RBPs is correlated with disease-free survival (DFS) in patients with TGCT. We assessed the specificity and sensitivity of 10-year survival status prediction using receiver operating characteristic (ROC) curve analysis, and successfully validated the results in multiple cohorts. Functional analysis was carried out to seek the possible mechanisms and pathways. We also examined the association between the model based on six RBPs and different clinical characteristics. A TGCT nomogram was established for recurrence prediction. Consensus clustering analysis was carried out to identify the clusters of TGCT with different clinical outcomes.

## Materials and Methods

### Patient Selection and Data Collection

The expression data and clinical information of RBPs in 156 patients with TGCT were downloaded from The Cancer Genome Atlas (TCGA) datasets,^1^ and the data of 165 healthy controls were obtained from Genotype-Tissue Expression (GTEx).^2^ We downloaded “TCGA-TGCT.survival.tsv.gz” and “TCGA-TGCT.htseq_fpkm.tsv.gz” from TCGA datasets. We further downloaded “gtex_RSEM_gene_fpkm.gz” and “GTEX_phenotype.gz” from the GTEx dataset and selected patients whose “primary site” is “Testis.” Patients with Patient data included a complete RBP expression profile and survival data for DFS. This study complied with TCGA publication guidelines and policies. No ethics approval was demanded for this study because data were obtained from online public resources.

In this study, we selected 1,542 RBPs for further analysis (Gerstberger et al., 2014). Adjusted false discovery rate (FDR) < 0.05 and absolute | log2 fold change| > 0.5 were chosen as the cut-off threshold. “Limma” package (3.46.0) was used for screening the differentially expressed genes (DEGs) between cancer samples and normal control samples.

### Protein–Protein Interaction (PPI) Network Construction and Module Analysis

In the current study, information on the protein–protein interaction (PPI) network of RBPs were obtained from the Search Tool for the Retrieval of Interacting Genes (STRING) database^3^ with interaction score >0.7, and 388 differentially expressed RBPs were chosen to constitute the PPI network. Plug-in Molecular Complex Detection (MCODE) was applied to detect densely connected regions in PPI networks, which was visualized by Cytoscape (version 3.8.0).^4^ The PPI networks were constructed using Cytoscape and the most significant module in the PPI networks was selected using MCODE. The criteria for selection were set as follows: maximum depth = 100, degree cut-off = 2, node score cut-off = 0.2, MCODE scores > 5 and K-score = 2.

### Construction of Prognostic Prediction Model

**FIGURE 1:**
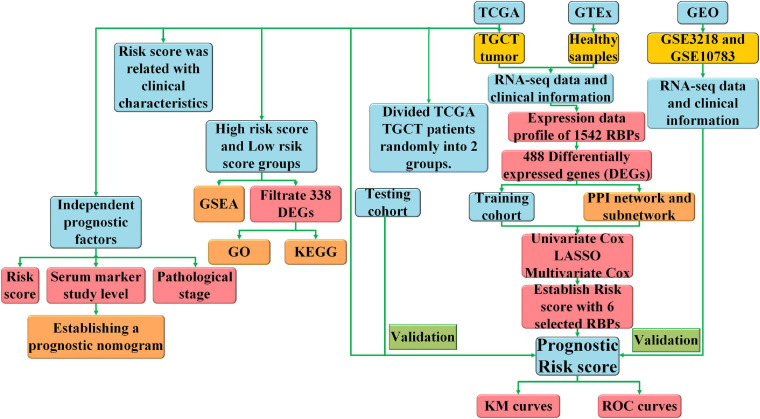
The flow chart of the study design and analysis.

Our work was designed and executed following the process presented in the flow chart (Figure 1) to find out the roles of RBPs in TCGT. All patients with TGCT in TCGA were randomly divided into two groups. Among 121 patients, 79 were selected as a training cohort and 42 cases were classified into a test cohort. We carried out Cox univariate analysis to identify possible prognostic RBPs based in the training cohort. Then, we excluded some genes highly correlated with one another using least absolute shrinkage and selection operator (LASSO) Cox regression algorithm to avoid overfitting the model. Ten RBPs were selected for further Cox multivariate proportional hazards regression analysis. Finally, six RBPs (*TRMT61A, POLR2J, DIS3L2, IFIH1, IGHMBP2*, and *NPM2*) were identified for the construction of risk score. Risk score was calculated through the following formula: Risk score = $\sum_{i=1}^{n}\text{coef}\,\left(i\right)\times x\,\left(i\right)$ where *n*, coef(*i*), and *x*(*i*) represent the number of genes, the coefficient of each gene, and the relative expression value of each gene selected by multivariate analysis, respectively. Patients were divided into high-risk and low-risk groups based on the median risk score of all samples. Kaplan–Meier curve and log-rank test were carried out to evaluate the relationship between DFS and risk score. The “glmnet,” “survival,” and “survminer” packages in R were used to construct the prognostic prediction model.

### Validation of Prognostic Signature

Patients in the test cohort were classified into high-risk and low-risk groups based on the same cut-off risk score. Kaplan–Meier curve and log-rank test were carried out to evaluate the relationship between DFS and risk score. Then, we validated the prediction accuracy of the prognostic model in the entire TCGA cohort. Kaplan-Meier curve and ROC were carried out. Chi-square test was performed to evaluate the association between clinicopathological parameters and the risk score in the entire TCGA cohort. Univariate and multivariate Cox regression analyses were used in the entire TCGA cohort to investigate whether risk score was an independent prognostic factor. The prognostic value of risk score stratified by clinicopathological parameters was further assessed. The “survival,” “survminer,” and “survivalROC” packages in R were used to carry out Kaplan-Meier and ROC analysis.

### Establishment and Validation of Nomogram for Prognosis Prediction

A nomogram for prognosis prediction was constructed using all independent prognostic factors (risk score, serum marker levels, and pathological stage). Calibration plots were used to investigate the calibration of the nomogram. The “rms,” “foreign,” and “survival” packages in R were used to establish and validate the nomogram.

### Functional Analysis

After dividing the patients with TGCT into two groups based on six-RBP risk score, we subsequently performed Gene Ontology (GO) and Kyoto Encyclopedia of Genes and Genomes (KEGG) pathway enrichment analyses of DEGs between the two groups using the “clusterProfiler” R package to extract the information of the enriched functions and pathways of the prognostic model based on six RBPs. GO analysis consists of three terms: biological process (BP), cellular component (CC), and molecular function (MF). FDR < 0.05 and | log2FC| > 0.5 were used as thresholds. Gene set enrichment analysis (GSEA) is a systematic method used to figure out whether hallmark gene sets predicted have statistically significant differences between two different groups (Subramanian et al., 2005). We carried out GSEA to analyze significant differences between the survival of the high-risk and low-risk groups in the entire TCGA cohort. Normalized *p* < 0.05 and FDR < 0.25 were considered significant differences.

### Consensus Clustering Analysis

The TGCT cohort was clustered into two groups according to the consensus expression of six selected RBPs using “Consensus Cluster Plus” in R to check out whether the expression levels of RBPs have prognostic value. Chi-square test was used to compare the distribution of serum markers, lymphovascular invasion, pathological stage, TMN stage, type, and age between two clusters. We carried out principal component analysis (PCA) to compare the transcriptional profile between clusters 1 and 2.

### External Validations of the Six-Gene Risk Score Using the Gene Expression Omnibus Database

The six-gene risk score was further verified using two gene expression profiles (GSE3218 and GSE10783) extracted from the Gene Expression Omnibus (GEO) database.^5^ We used the following terms to search the eligible GEO datasets: (“Germ Cell Tumors” or “Testicular Germ Cell Tumor”) and (“outcome” or “prognosis”) and (“male”). The eligible studies met the following inclusion criteria: (Tsili et al., 2019) reported research on patients with TCGT; (Zhang et al., 2018) provided sufficient clinical data to calculate overall survival (OS), DFS, or progression-free survival; and (Diamantopoulos and Kortsaris, 2010) provided gene expression profiles of TGCT. Only peer-reviewed studies were deemed eligible for inclusion. Conference abstracts and case reports were excluded. After searching the databases, the data source of the references was examined to avoid duplicates. Data from the most complete study was extracted when duplicates in study population were found. The “sva” package was used to remove batch effects and other needless variables. Overall, 108 samples from GSE3218 and GSE10783 were used as the independent external validation cohort. Kaplan-Meier curve and log-rank test were carried out to evaluate the relationship between OS and risk score.

### Statistical Analysis

All statistical data and figures were analyzed using R 4.0.0. We assessed the different expression of RBPs between the control and TGCT groups by Wilcoxon’s test. Chi-square test was performed to evaluate the association between risk score and clinicopathological parameters. Univariate and multivariate Cox regression analyses were used in the entire TCGA cohort to investigate whether risk score is an independent prognostic factor. ROC curve and the area under the ROC curve (AUC) were used to evaluate the prognostic ability of risk score using the package of “survivalROC” in R. All statistical results with *p* < 0.05 were considered statistically significant.

## Results

### Differentially Expressed RBPs in TGCT

**FIGURE 2:**
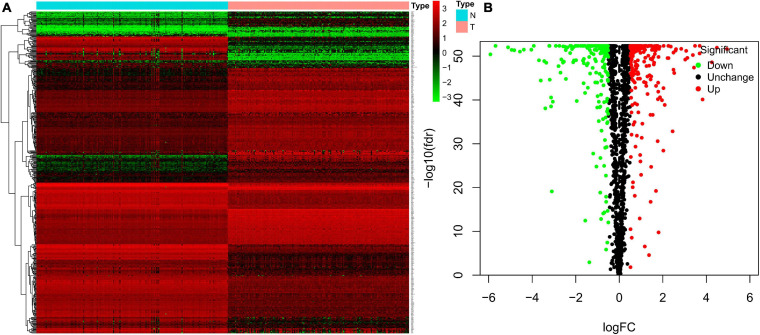
Heatmap and volcano plots of TGCT patients from TCGA. Heatmap **(A)** and volcano **(B)** plots were generated with FDR < 0.05 and | log2FC| > 0.5, using the data of differentially expressed RBPs in TGCT downloaded from TCGA and GTEx. TGCT, Testicular germ cell tumors; TCGA, The Cancer Genome Atlas; RBPs, RNA-binding proteins; GTEx, Genotype-Tissue Expression; N, Normal controls from healthy individuals; T, tumor samples of testicular germ cell tumors.

We analyzed the expression profiles of RBPs between 156 tumor samples and 165 healthy controls healthy individuals from TCGA and GTEx. We screened 489 DEGs, including 287 significantly up-regulated RBPs and 222 down-regulated RBPs. All the DEGs were identified based on | log2FC| > 0.5 and FDR < 0.05. Heatmap and volcano plot (Figure 2) were also constructed to visualize the expression patterns of DEGs, in which red and green colors represent relatively high and low expression levels, respectively.

### PPI Network Among Differentially Expressed RBPs

**FIGURE 3:**
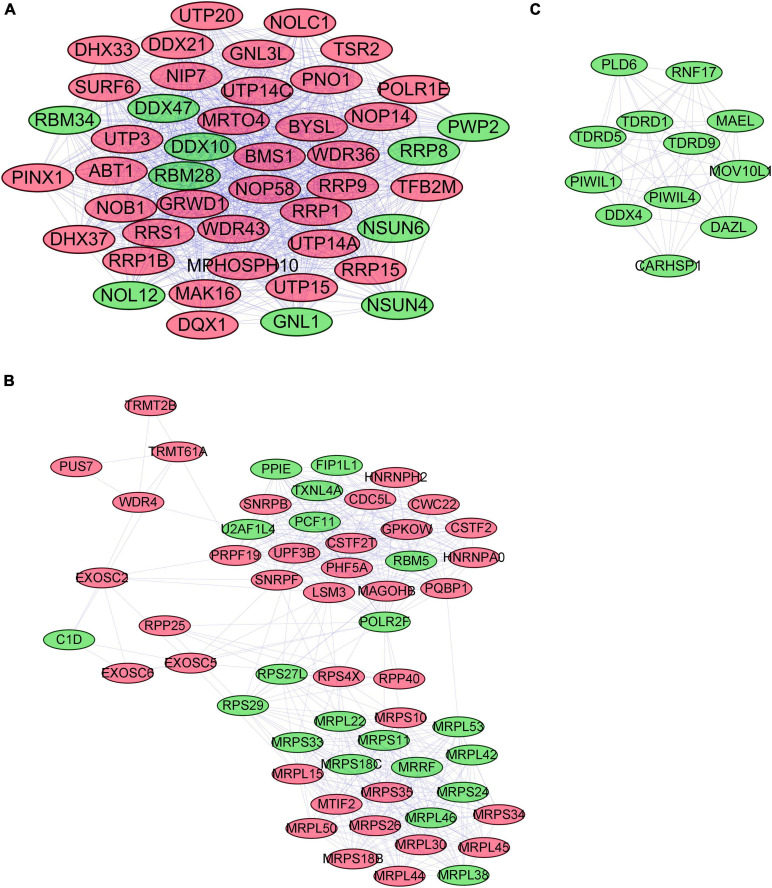
The most critical modules in the PPI network of differentially expressed RBPs **(A–C)**. Significant modules were obtained from the PPI network using MCODE of Cytoscape. Modules with red color and green color relatively represent high expression and low expression levels of RBPs. The criteria for selection were set as follows: Max depth = 100, degree cut-off = 2, Node score cut-off = 0.2, MCODE scores > 5, and K-score = 2. PPI, protein–protein interactions; RBPs, RNA-binding proteins; MCODE, Molecular Complex Detection.

We used STRING to establish the PPI network and further explore the correlation and interaction between the differentially expressed RBPs. Cytoscape was also applied to visualize the interaction network. The most meaningful modules were selected and are shown in Figure 3, in which red and green colors meant relatively high and low gene expression, respectively.

### Construction of RBP-Related Risk Score in the TCGA Training Cohort and Validation of the Risk Score in the TCGA Test Group

**FIGURE 4:**
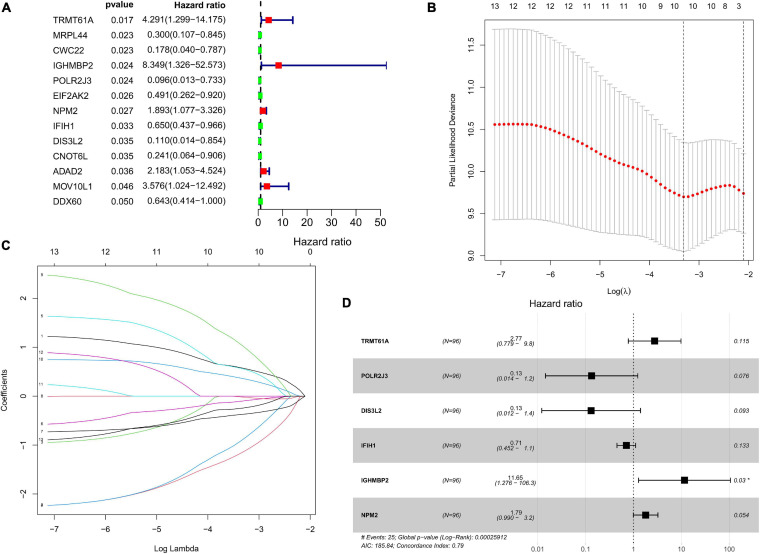
Prognostic value of RBPs in TCGA TGCT training cohort. **(A)** Cox univariate analysis of RBP genes in the training cohort. **(B,C)** Multivariate Cox regression via LASSO is presented, and ten candidate RBPs were selected in training cohort. **(D)** Forrest plot of the multivariate Cox regression analysis in TGCT. TGCT, Testicular germ cell tumors; TCGA, The Cancer Genome Atlas; RBPs, RNA-binding proteins; LASSO, last absolute shrinkage and selection operator.

**FIGURE 5:**
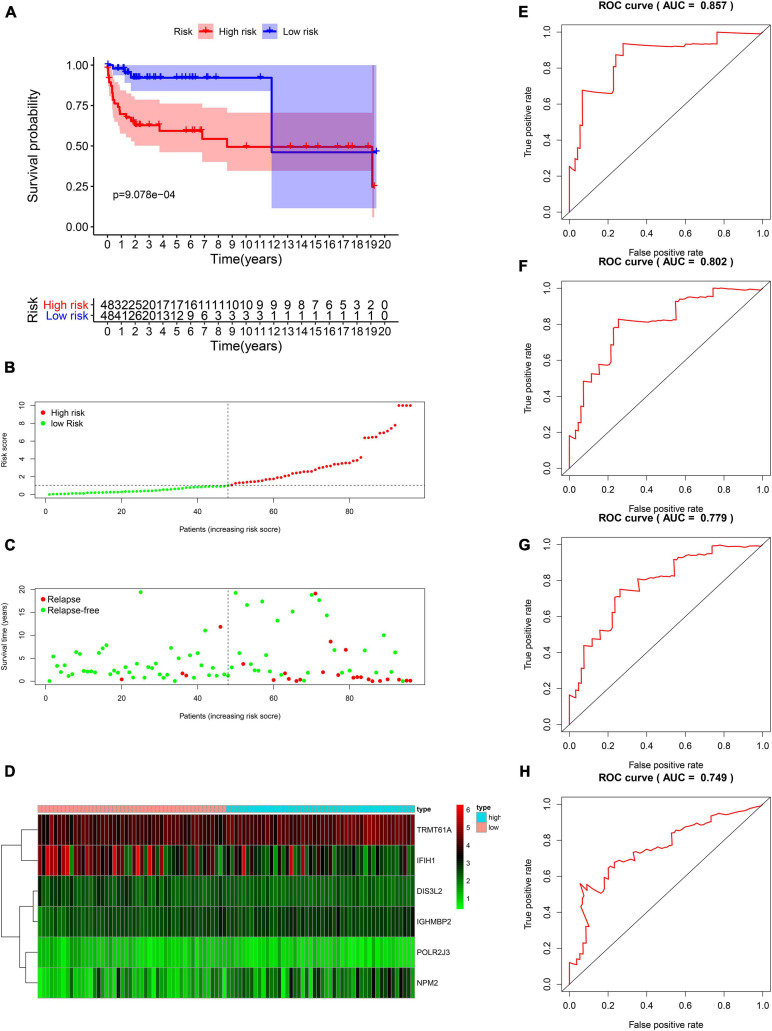
Survival analysis based on the risk model in the training group. **(A)** Kaplan–Meier survival curve analysis of DFS in the high-risk and low-risk TGCT patients in the training group, marked as red line and blue line separately. **(B–D)** Risk score, distribution of survival status between the high-risk and low-risk groups, and expression heat maps of six RBPs. **(E–H)** Time-dependent ROC curve analyses was conducted and AUC values were calculated for 1-, 3-, 5-, and 10-year DFS in the TGCT training cohort. TGCT, Testicular germ cell tumors; RBPs, RNA-binding proteins; DFS, disease-free survival; ROC, Receiver operating characteristic curve; AUC, the area under the ROC curve; High, high-risk group; Low, low-risk group.

**FIGURE 6:**
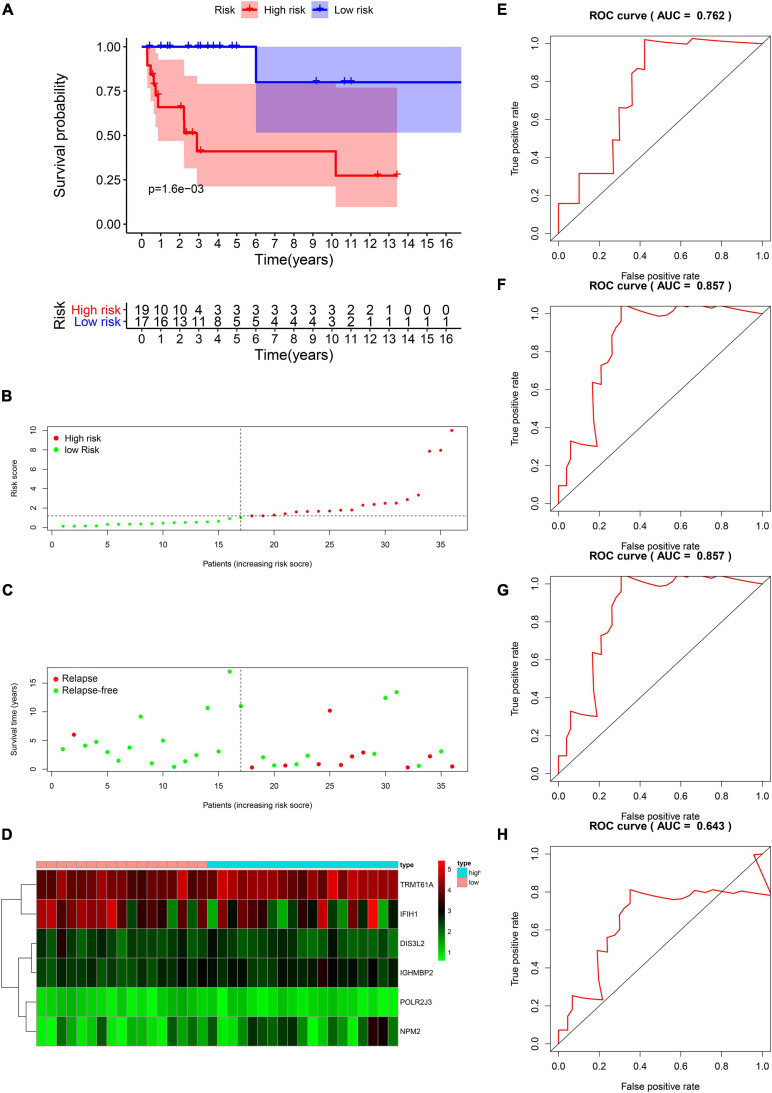
Validation of the prognostic value of the risk model in the test group. **(A)** Kaplan–Meier survival curve analysis of DFS in the high-risk and low-risk TGCT patients in the test group, marked as red line and blue line separately. **(B–D)** Risk score, distribution of survival status between the high-risk and low-risk groups and expression heat maps of six RBPs. **(E–H)** Time-dependent ROC curve analyses was conducted and AUC values were calculated for 1-, 3-, 5-, and 10-year DFS in the TGCT test cohort. TGCT, Testicular germ cell tumors; DFS, disease-free survival; RBPs, RNA-binding proteins; TGCT, Testicular germ cell tumors; ROC, Receiver operating characteristic curve; AUC, the area under the ROC curve; High, high-risk group; Low, low-risk group.

The DFS data of 132 patients with TGCT were obtained from TCGA and randomly divided into the training and test groups. In the training group, we conducted univariate Cox regression based on DFS and identified 13 genes that strongly indicated TGCT prognosis and were significantly related to DFS (*p* < 0.05, Figure 4A). Then, we used LASSO regression to avoid overfitting (Figures 4B,C). We finally targeted six key genes (*TRMT61A, POLR2J, DIS3L2, IFIH1, IGHMBP2*, and *NPM2*) that met the modeling requirement by multivariate Cox regression analysis (Figure 4D). Each gene expression value was matched with its relevant coefficient, and the risk scores of the training cohort were calculated using the formula: Risk score = (*TRMT61A* expression) × (1.01815998520062) + (*POLR2J3* expression) × (−2.01356263 039402) + (*DIS3L2* expression) × (−2.03760675664572) + (*IFIH1* expression) × (−0.34441879625446) + (*IGHMBP2* expression) × (2.45508579120534) + (*NPM2* expression) × (0.582848422396182). Patients in the training group were subdivided into high- and low-risk groups according to the median risk score. Kaplan–Meier survival curve analysis revealed that the high-risk group has worse prognosis than the low-risk group (*p* < 0.001, Figure 5A). Based on the prognostic model, the survival status or prognosis of patients with TGCT became worse, that is, the number of dead patients increased as the risk score increased (Figures 5B,C). The expression patterns of the six risk RBPs in the high-risk and low-risk groups are shown in the a heatmap in Figure 5D. Time-dependent ROC analysis was applied to assess the predictive efficiency of the model. The AUCs were 0.857, 0.802, 0.779, and 0.749 at 1, 3, 5, and10 years, respectively (Figures 5E–H). The results indicated that the model is sensitive and has fair accurance for prognosis prediction. We validated the prognostic models in the test group and proved that the model has the same predictive function as that in the training group (Figure 6). In the test group, the high-risk group has worse prognosis compared with the low-risk group in Kaplan–Meier survival curve analysis (*p* < 0.01, Figure 6A). Time-dependent ROC curve was made, and the AUCs were 0.762 at 1 years, 0.857 at 3 and 5 years, and 0.643 at 10 years (Figures 6E–H).

### Validation of Risk Score in the Entire TCGA Cohort

**FIGURE 7:**
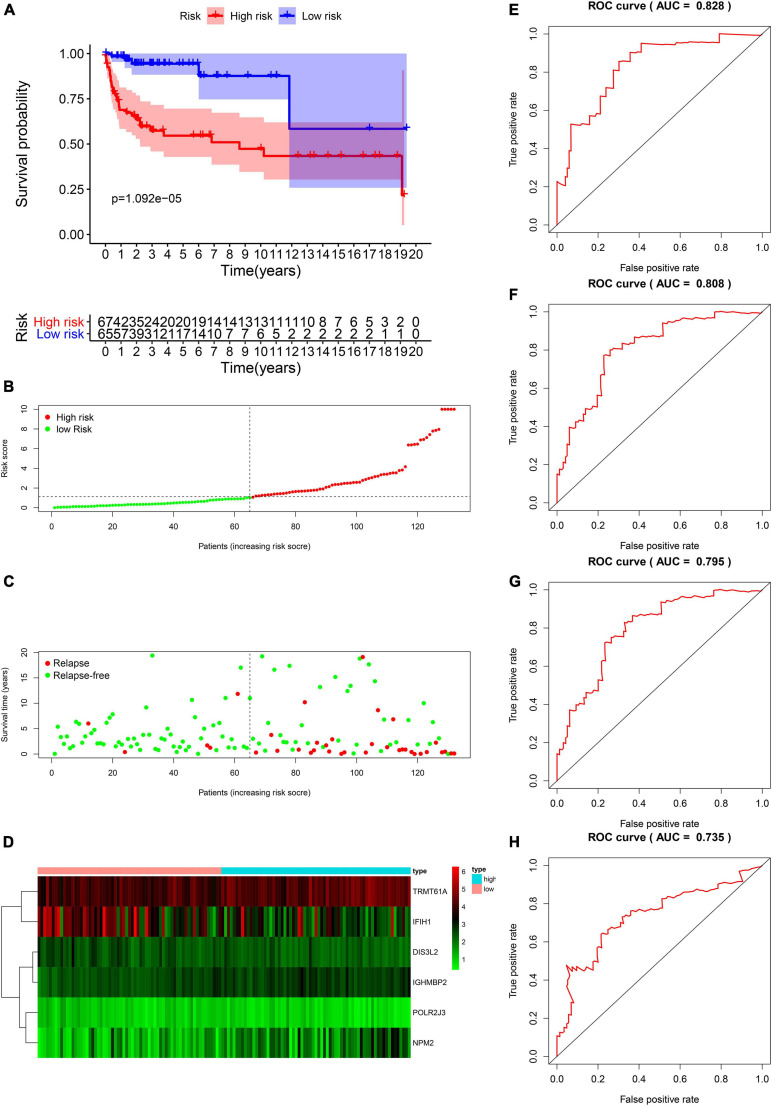
Validation of the prognostic value of the risk model in the entire TCGA cohort. **(A)** Kaplan–Meier survival curve analysis of DFS in the high-risk and low-risk TGCT patients in the entire TCGA cohort, marked as red line and blue line separately. **(B–D)** Risk score, distribution of survival status between the high-risk and low-risk groups, and expression heat maps of six RBPs. **(E–H)** Time-dependent ROC curve analyses was conducted and AUC values were calculated for 1-, 3-, 5-, and 10-year DFS in the entire TCGA TGCT cohort. TGCT, Testicular germ cell tumors; TCGA, The Cancer Genome Atlas; DFS, disease-free survival; ROC, Receiver operating characteristic curve; AUC, the area under the ROC curve; TGCT, Testicular germ cell tumors.

We proved that risk score is valuable in TGCT prognosis prediction in the train and test groups. Next, the model was validated using the entire TCGA cohort. The Kaplan–Meier plots reveal that patients in the high-risk groups exhibited worse prognosis than those in the low-risk group (*p* < 0.001, Figure 7A). The patients with TGCT from the entire TCGA cohort have worse outcomes as indicated by the increased number of relapsing patients as the risk score increased (Figures 7B,C). The heatmap of the six key genes shows that the expression of TRMT61A is high in the high-risk and low-risk group, whereas *DIS3L2, IGHMBP2, POLR2J3*, and *NPM2* have low expression in the two groups (Figure 7D). IFIH1 has relatively lower expression in the high-risk group than in the low-risk group. Time-dependent ROC curve was constructed, and the AUCs were 0.828 at 1 years, 0.808 at 3 years, 0.795 at 5 years, and 0.735 at 10 years (Figures 7E–H). Meanwhile, we also observed that comparing with each gene alone, the six genes-based risk score showed better prognostic value in 1-year (AUC = 0.828, Supplementary Figure 1A), 3-year (AUC = 0.808, Supplementary Figure 1B), 5-year (AUC 0.795, Supplementary Figure 1C), and 10-year DFS prediction (AUC = 0.735, Supplementary Figure 1D).

### Prognostic Signature-Based Risk Score Was an Independent Prognostic Factor in the TCGA TGCT Cohort

**FIGURE 8:**
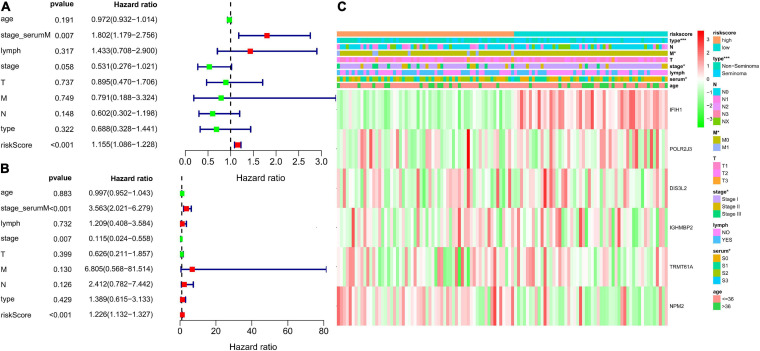
Analysis for evaluating the independent prognostic value of the risk score. Forrest plot of univariate **(A)** and multivariate **(B)** Cox regression analysis of risk score, age, serum markers, lymphovascular invasion, TNM stage, disease type, and stage. **(C)** Significant differences were found for the disease type, M stage, serum markers, and stage between high- and low-risk group. *p < 0.05, **p < 0.01, and ***p < 0.001 compared with the low-risk group.

Patient information, such as risk score, age, stage based on serum marker study levels, lymphovascular invasion, TNM stage, pathological stage, and pathological type, were extracted. Univariate and multivariate Cox regression analysis were conducted. The results presented as forest maps obviously indicate that the risk score correlated independently with DFS in univariate [hazard ratio (HR) = 1.155, confidence interval (CI) = 1.086−1.228, ^∗∗∗^*p* < 0.001], and multivariate Cox regression analyses (HR = 1.226, 95% CI = 1.132−1.327, ^∗∗∗^*p* < 0.001; Figures 8A,B). Stage based on serum markers is also an independent predictor as validated by univariate (HR = 1.802, 95% CI = 1.179−2.756, ^∗∗^*p* = 0.007) and multivariate Cox regression analyses (HR = 3.563, 95% CI = 2.021−6.279, ^∗∗∗^*p* < 0.001). In addition, pathological stage was validated in multivariate Cox regression analysis (HR = 0.115, 95%CI [0.024−0.558], ^∗∗^*p* = 0.007). The heat map of the expression levels of the six key RBPs in the high- and low-risk groups patients in the TCGA cohort is presented in Figure 8C. Clinicopathological parameters, namely, pathological type (^∗∗∗^*p* < 0.001), pathological stage (^∗^*p* < 0.05), and higher M stage (^∗^*p* < 0.05), were significantly different between the high- and low-risk groups (Figure 8C).

**FIGURE 9:**
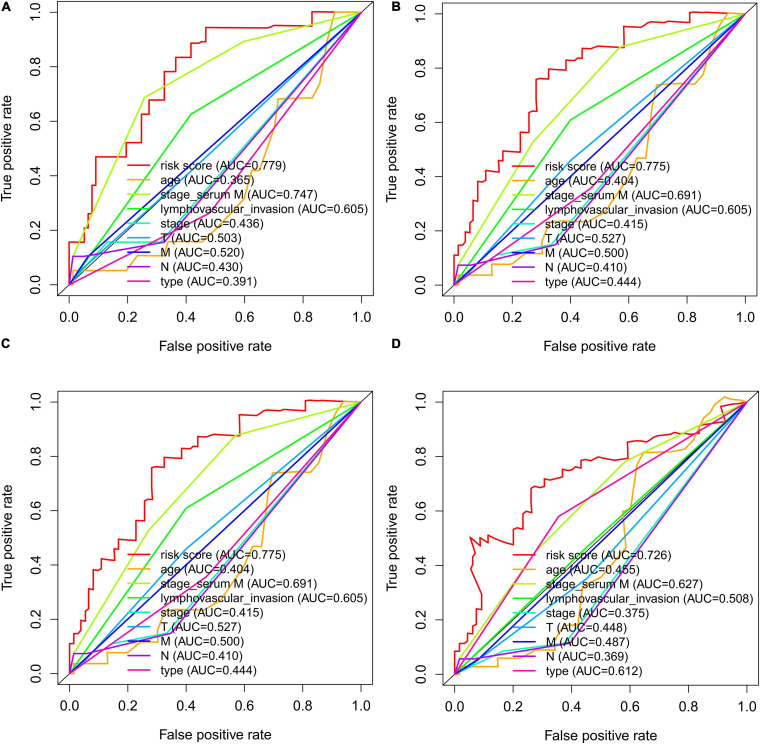
The time-dependent ROC curves for risk score, age, serum markers, lymphovascular invasion, TNM stage, disease type, and stage combining with 1- **(A)**, 3- **(B)**, 5- **(C)**, and 10- **(D)** year DFS in TCGA TGCT cohort, respectively. DFS, disease-free survival; ROC, Receiver operating characteristic curve; TCGA, The Cancer Genome Atlas; TCGA, The Cancer Genome Atlas; DFS, disease-free survival; ROC, Receiver operating characteristic curve; TGCT, Testicular germ cell tumors.

Time-dependent ROCs were applied to assess the prognostic accuracy of factors, including risk score, age, serum marker study levels, lymphovascular invasion, TNM stage, pathological stage, and type in patients with TGCT (Figure 9). Compared with other factors, risk score manifested superior accuracy in predicting 1-year (AUC = 0.779, Figure 9A), 3-year (AUC = 0.775, Figure 9B), 5-year (AUC = 0.775, Figure 9C), and 10-year survival (AUC = 0.726, Figure 9D). Stage based on serum marker study levels was only inferior to risk score but also showed great accuracy in predicting 1-year (AUC = 0.747, Figure 9A), 3-year (AUC = 0.691, Figure 9B), 5-year (AUC = 0.691, Figure 9C), and 10-year survival (AUC = 0.627, Figure 9D).

### The Survival Difference Between High- and Low-Risk Group Stratified by Clinicopathological Parameters in the TCGA TGCT Cohort

**FIGURE 10:**
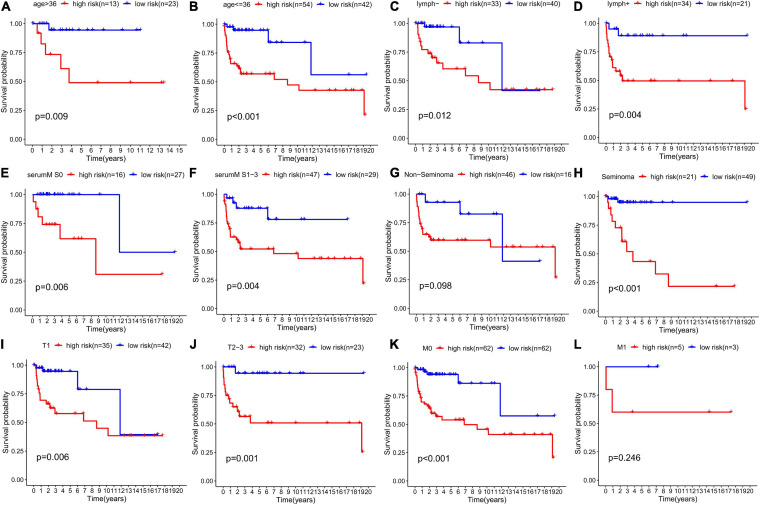
Prognostic value of the risk score in TGCT patients classified into specific cohorts. Kaplan–Meier survival curve of DFS for patients with **(A)** age >36, **(B)** age ≤36, **(C)** no lymphovascular invasion, **(D)** lymphovascular invasion, **(E)** serum marker study levels within normal limits, **(F)** serum marker study levels beyond the normal limits, **(G)** non-seminoma, **(H)** seminoma, **(I)** T1 stage, **(J)** T2-3 stage, **(K)** M0 stage, and **(L)** M1 stage in the high-risk (red line) and low-risk (blue line) TGCT patients. TGCT, Testicular germ cell tumors.

We grouped the patients by clinical characteristics to figure out whether risk score has prognostic value in various clinicopathological parameters. Kaplan–Meier curves were constructed to show that patients in the high-risk group with clinical characteristics, such as age ≥36 (*p* = 0.009, Figure 10A), age ≤36 (*p* < 0.001, Figure 10B), no lymphovascular invasion (*p* = 0.012, Figure 10C), lymphovascular invasion (*p* = 0.004, Figure 10D), serum tumor marker levels within normal limits (*p* = 0.006, Figure 10E), serum tumor marker levels beyond normal limits (*p* = 0.004, Figure 10F), seminoma (*p* < 0.001, Figure 10H), T1 stage (*p* < 0.006, Figure 10I), T2–3 stage (*p* = 0.001, Figure 10J), M0 stage (*p* < 0.001, Figure 10K), had significantly lower DFS rate than those in the low-risk group. However, no significant difference was found in patients diagnosed with non-seminoma (*p* < 0.098, Figure 10G) and M1 stage (*p* = 0.246, Figure 10L).

**FIGURE 11:**
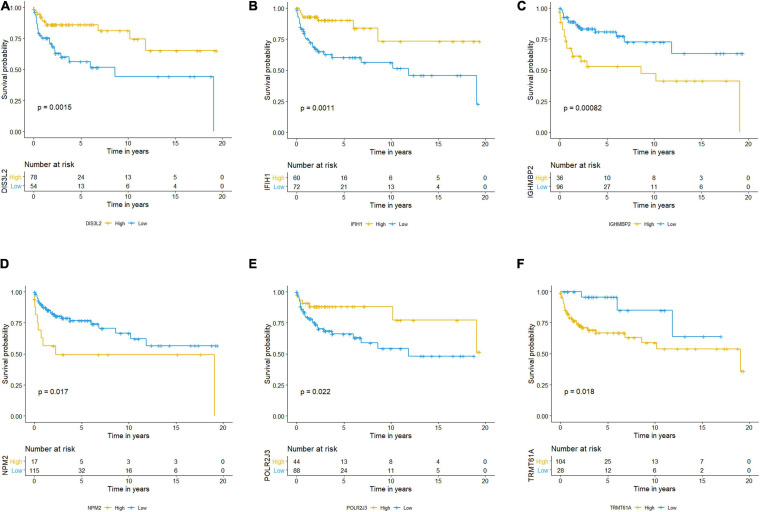
Kaplan–Meier survival analysis and the expression profiles of the six RBPs. **(A–F)** Kaplan–Meier plots showed distributions in survival probabilities of six RBPs in high-risk (yellow line) and low-risk (blue line) TGCT patients. TGCT, Testicular germ cell tumors; RBPs, RNA-binding proteins.

The Kaplan–Meier plots of the risk scores based on the six RBPs are exhibited in Figure 11. The patients with TCGT in the low-risk group who had high *DIS3L2, IFIH1*, and *POLR2J3* expression and low *TRMT61A, IGHMBP2*, and *NPM2* expression presented better prognosis than those in the high-risk group, and differences were all statistically significant. Therefore, these six genes could play a prognostic role in TCGT prognosis prediction.

### Establishment of TGCT DFS Prediction Nomogram

**FIGURE 12:**
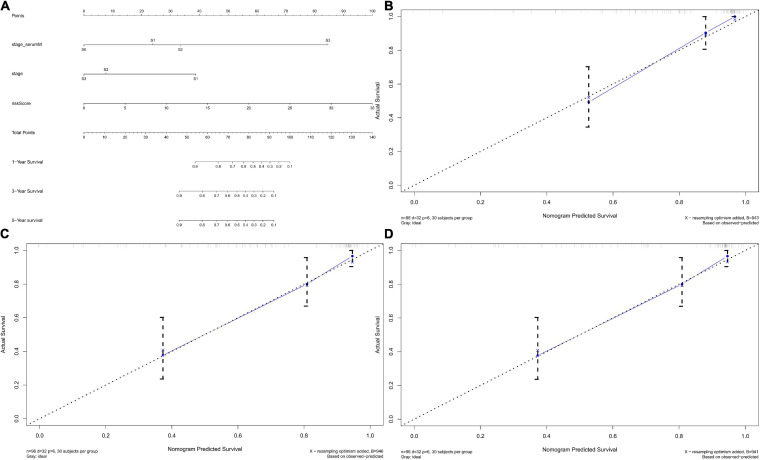
Nomograms predicting survival probability of TGCT patients in TCGA. **(A)** Nomogram to predict 1-, 3-, and 5-year DFS. **(B–D)** Calibration plots of 1-, 3-, and 5-year DFS for nomograms. TCGA, The Cancer Genome Atlas; DFS, disease-free survival; TGCT, Testicular germ cell tumors.

We established the nomogram predictive model to better predict the probability of disease progression by integrating independent factors associated with prognosis (serum marker study levels, stage, and risk score; Figure 12A). We could predict the 1-year (Figure 12B), 3-year (Figure 12C), and 5-year (Figure 12D) survival rates of TGCT patients respectively according to the total points of all risk factors by quantifying the variables above as numeric scores and adding them up. We also constructed calibration plots to manifest that the predicted outcomes were consistent with the observed outcomes. The performance of the nomogram in accurately predicting the 1-, 3-, and 5-year survival of diagnosed patients was proven in this study.

### Identification of Signaling Pathways Related to Risk Score by GO, KEGG, and GSEA

**FIGURE 13:**
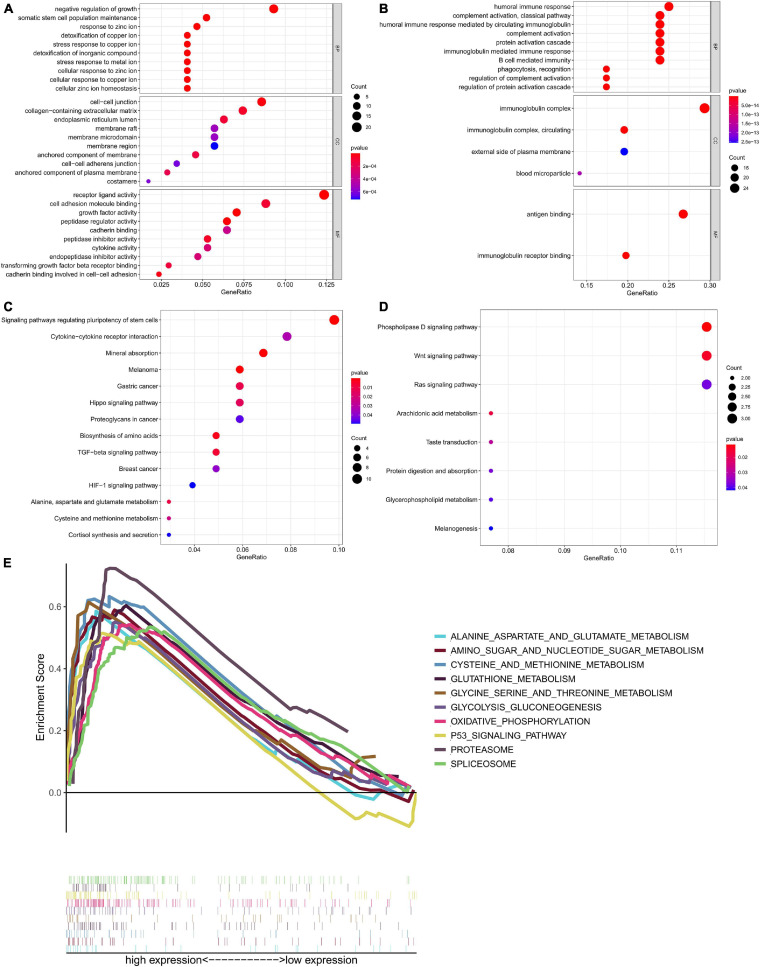
Functional annotation of the differentially expressed genes between high risk and low risk groups based on six-RBP risk score of TGCT in TCGA cohort. Bubble plots showed enrichment of **(A)** GO terms associated with up-regulated genes and **(B)** GO terms associated with down-regulated genes. Bubble plots showed enrichment of **(C)** KEGG pathway associated with up-regulated genes and **(D)** KEGG pathway associated with down-regulated genes. **(E)** GSEA analysis showed the top ten most significantly enriched signaling pathways in high-risk score subgroup. GO, Gene Ontology; KEGG, Kyoto Encyclopedia of Genes and Genomes.

We divided the 132 patients into high-risk and low-risk groups based on risk score and identified 338 DEGs based on the criteria | log2FC| > 1 and FDR < 0.05. We aimed to illuminate the biological functions and pathways related to DEGs between high-risk and low-risk groups by performing GO and KEGG pathway enrichment analyses. As illustrated in Figure 13A, The ten most significant BP terms related with the up-regulated RBPs include “negative regulation of growth,” “somatic stem cell population maintenance,” “response to zinc ion,” “detoxification of copper ion,” “stress response to copper ion,” “stress response to metal ion,” “cellular response to zinc ion,” “cellular response to copper ion,” and “cellular zinc ion homeostasis.” In Figure 13B, the 10 most significant BP terms related with down-regulated RBPs include “humoral immune response,” “complement activation, classical pathway,” “humoral immune response mediated by circulating immunoglobulin,” “complement activation,” “protein activation cascade,” “immunoglobulin mediated immune response,” and “B cell mediated immunity.” Likewise, GO CC terms “cell-cell junction,” “collagen-containing extracellular matrix,” “endoplasmic reticulum lumen,” “membrane raft,” “membrane microdomain,” “immunoglobulin complex,” “immunoglobulin complex, circulating,” “external side of plasma membrane,” and “blood microparticle” were enriched. GO MF terms revealed that highly expressed RBPs were enriched in “receptor ligand activity,” “cell adhesion molecule binding,” “growth factor activity,” “peptidase regulator activity,” “cadherin binding,” “peptidase inhibitor activity,” “cytokine activity,” “transforming growth factor beta receptor binding,” and “cadherin binding involved in cell–cell adhesion,” and the lowly expressed RBPs were enriched in “antigen binding” and “immunoglobulin receptor binding.”

The significant pathways related with the DEGs listed in Figures 13C,D were analyzed by KEGG. The pathways include “signaling pathways regulating pluripotency of stem cells,” “cytokine–cytokine receptor interaction,” “mineral absorption,” “melanoma,” “proteoglycans in cancer,” “biosynthesis of amino acids,” “TGF-beta signaling pathway,” “breast cancer,” “HIF-1 signaling pathway,” Pathways such as “phospholipase D signaling pathway,” “Wnt signaling pathway,” “Ras signaling pathway,” “arachidonic acid metabolism,” and “protein digestion and absorption” were negatively related to risk score.

Gene set enrichment analysis was performed to further investigate the possible mechanisms leading to different outcomes in the high-risk group and the low-risk group, and we did GSEA based on the patients in two groups (Figure 13E). “Alanine aspartate and glutamate metabolism,” “amino sugar and nucleotide sugar metabolism,” “cysteine and methionine metabolism,” “glutathione metabolism,” “glycine serine and threonine metabolism,” “glycolysis gluconeogenesis,” “oxidative phosphorylation,” “p53 signaling pathway,” “proteasome,” and “spliceosome” were the enriched pathways in the high-risk group. The results of GSEA demonstrated that most of the DEGs in the high-risk group were enriched in cancer pathways and metabolism related pathways.

### Consensus Clustering of Risk Score Based on Six Independent Prognostic RBPs Identified Two Clusters of TGCT With Different Clinical Outcomes

**FIGURE 14:**
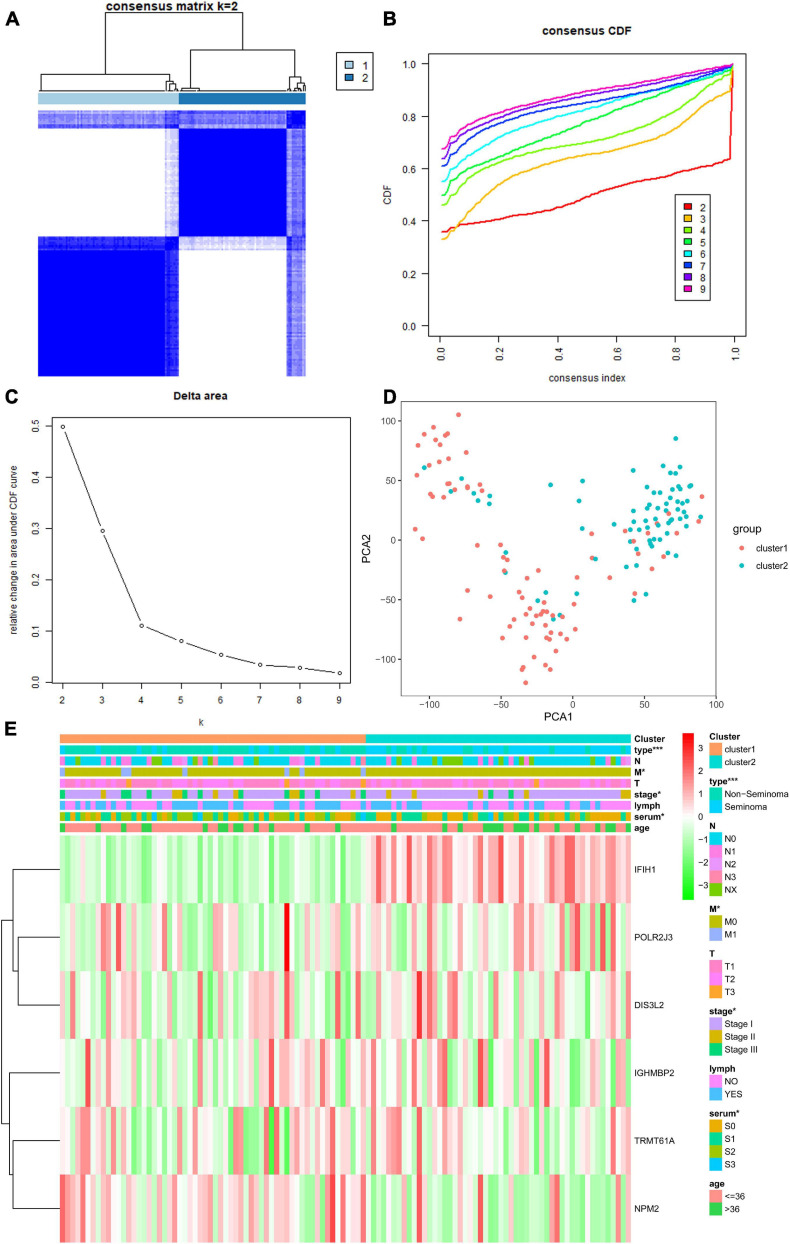
Differential clinical characteristics of TGCT patients in the two different clusters. **(A)** Based on the expression similarity of RBPs, the TCGA TGCT cohort was separated into two distinct clusters when *k* = 2. **(B,C)** Consensus CDF and relative change in area under CDF curve for *k* = 2–9. **(D)** Principal component analysis based on two clusters. **(E)** Significant difference was observed for the type, grade, stage, and serum marker study levels between cluster 1 and cluster 2. CDF, clustering cumulative distribution function. *p < 0.05, **p < 0.01, and ***p < 0.001 compared with the cluster2 group. TGCT, Testicular germ cell tumors; RBPs, RNA-binding proteins; TCGA, The Cancer Genome Atlas.

We selected k = 2 as the most appropriate selection to divide the TGCT patient cohort into two clusters, namely, cluster 1 and cluster 2, according to the expression similarity of risk score based on independent prognostic RBPs and the criteria for selecting the number of clusters (Figures 14A–C). PCA was carried out to compare the transcriptional profiles of clusters 1 and 2, and substantial distinction was observed between the two subgroups (Figure 14D). The assessment of the correlation between the clusters and clinicopathological features showed significant differences in type (^∗∗∗^*p* < 0.001), M stage (^∗^*p* < 0.05), pathological stage (^∗^*p* < 0.05), and serum markers study levels (^∗^*p* < 0.05, Figure 14E).

### External Validations of the Six-Gene-Based Risk Score Using the GEO Database

**FIGURE 15:**
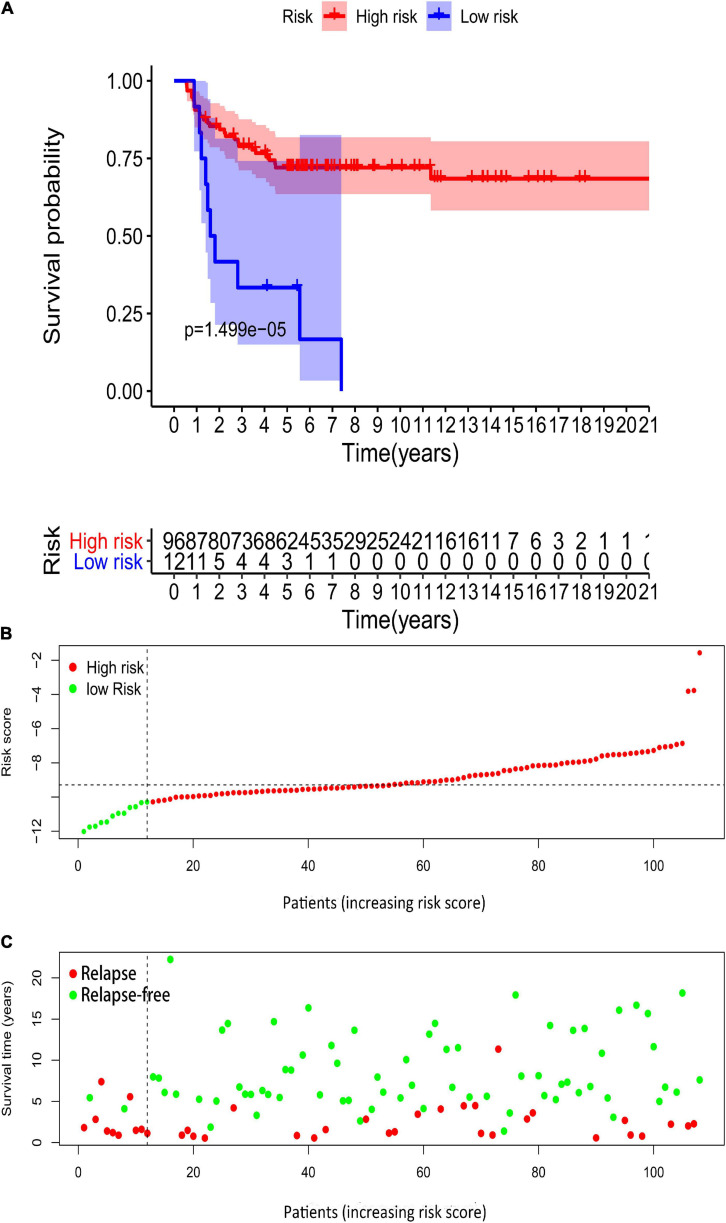
Validation of the prognostic value of the risk model in GSE3218 and GSE10783 datasets. **(A)** Kaplan–Meier survival curve analysis of OS in high-risk score and low-risk score TGCT patients in GSE3218 and GSE10783 datasets, marked as red line and blue line separately. **(B,C)** Risk score, distribution of survival status between the high-risk and low-risk groups, and expression heat maps of six RBPs. OS, overall survival; TGCT, Testicular germ cell tumors; TCGA, The Cancer Genome Atlas; RBPs, RNA-binding proteins.

We downloaded GSE3218 and GSE10783 datasets from the GEO database to validate the risk assessment formula. However, the risk score applied was inconsistent with the prognostic models we constructed previously. Higher risk group showed a better overall survival than low risk group (*p* = 0.00001499, Figure 15A). In terms of survival status the patients in high-risk cohorts have better OS than those in low-risk cohorts (Figures 15B,C). The reason for this adverse result is that the samples in GSE3218 and GSE10783 are mostly from patients with non-seminoma germ cell tumors (76 non-seminoma germ cell tumors, 15 embryonal carcinoma, 15 mature teratomas, 10 yolk sac tumors, 2 choriocarcinomas, 17 seminomas). In Figure 10, we found that this RBP-related risk score did not apply to patients with non-seminoma germ cell tumors. However, we cannot find other suitable GEO dataset for external validations.

## Discussion

In our current study, we screened six RBP genes with differential expression in patients with TGCT. Based on univariate and multivariate Cox analyses, we finally targeted six prognosis-related RBPs to build the risk-scoring system. The prognosis prediction ability of the system underwent full verification in the test group, the entire TCGA cohort, and the GEO database. We observed that most of the numerical values of AUCs, which were calculated based on the risk scoring system in training group, test group, and entire TCGA cohort, were between 0.7 and 0.9, indicating that the systems showed fair or good performance to predict the DFS of patients with TGCT according to the traditional academic point system for classifying the accuracy of a diagnostic test.^6^ We also discovered that risk-scoring system and serum marker levels are important independent predictors of prognosis for patients with TGCT. The constructed nomogram was found reliable for clinical application.

After integrating information from the risk-score system with clinical characteristics, we found that the model performed well in predicting patients with different clinical characteristics except for patients with non-seminoma and M1 stage. These parameters represent early tumor stage and better survival outcome, which means that model could function well in predicting the survival of patients with relatively better conditions. We noticed that patients with TGCT have relatively better outcomes compared with patients with other malignant neoplasms, and more than 95% of patients diagnosed with early stage TGCT can be cured (Lakomý et al., 2009). Researchers found that more than 80% of patients with TGCTs undergo conventional cisplatin-based chemotherapy could be cured, even at highly advanced stage, and thus exhibited good prognosis in TGCT (Einhorn, 2002).

The serum markers of TGCT, including AFP, hCG, and LDH, play crucial roles compared with other solid organ malignancies. A stage categorization of serum tumor markers stage category was created according to the levels of these serum markers. Markers with higher expression levels could predict worse TGCT outcome (Barlow et al., 2010). Based on our model, DFS decreased as risk scores rose in patients with TCGT who had serum marker levels within normal limits or beyond normal limits. We proposed an assumption that combination of the model and serum tumor markers may show better survival predicting function when the numerical values of traditional serum tumor markers of TCGT were within normal limits. AUCs of the risk score were all greater than 0.7, which implied its fair accuracy in prognosis prediction based on the above guidelines. Besides, the numerical values of AUCs were higher than other factors, including serum marker levels, which suggested the model might serve as a better prognostic indicator.

We noticed that risk score seems higher in patients diagnosed with non-seminoma subtype or age <36 years old. This finding is reasonable because TGCTs are considered one of the most common malignancies in young adult males at the reproductive age (Hayes-Lattin and Nichols, 2009). However, Terbuch et al. found among metastatic TGCT cases that although higher age is associated with worse OS, it is not correlated with worse DFS or higher disease progression risk. Elderly patients could also gain high cure rates as younger patients if they tolerate risk-adapted chemotherapy; thus, age might not remarkably affect the prognosis of metastatic TGCT cases (Terbuch et al., 2019).

Gene Ontology analysis results showed that BP terms concern chemical reactions on metallic ion. However, limited research looked into whether metallic ion plays a part in TGCT. Togawa et al. observed that paternal exposure to Cr might elevate the risk of TGCT in offspring. Although the evidence is limited (Togawa et al., 2016), more studies are needed to confirm whether environmental exposure to metal ion is a pathogenic factor of TGCT. KEGG analysis was conducted on basis of differentially expressed RBPs between “high-” and “low-risk score.” “Signaling pathways regulating pluripotency of stem cells” was the most significantly enriched pathway. Differentiation arrest in male primordial germ cells during fetal development (the precursors of male germ line) is deemed as an origin of TGCT. SSC niche along with factors in the microenvironment of SSC might promote the development of some TGCT; hence, stem cells might play an important role in TGCT pathogenesis (Silván et al., 2013). Embryonal carcinomas (ECs), as a type of non-seminoma, often differentiates into teratoma, yolk sac tumor, and choriocarcinoma for its pluripotency (Dixon and Moore, 1953; Pierce and Abell, 1970). KLF4, overexpressed in seminoma cells, prevents differentiation and maintains proliferation and pluripotency during germ cell tumorigenesis; therefore, KLF4 may be vital in the neoplastic transformation of testicular stem cells. Similarly, stem cell factors, such as NANOG (Nettersheim et al., 2011) and OCT4 (Cheng et al., 2007), serve as markers in malignant human germ cell development. “Cytokine–cytokine receptor interaction pathways” were explored in previous studies in TGCT and ECs. CAR-T cells, considered effective as an immunotherapy for ECs, could eliminate EC cells via a cell–cell contact-dependent Fas/FasL interaction. Fas/FasL interaction between tumor cells and CAR-T cells could be utilized for tumor escape reduction via elevating heterogeneous antigen expression or improving CAR T-cell antitumor activity (Hong et al., 2018). Guido et al. found that estradiol could induce *PTEN* gene expression by binding to ERβ. ERβ/PTEN signaling induces cell death in seminoma cell lines by autophagy and necroptosis (Guido et al., 2012). Notably, the interaction between cytokines and their receptors were often discussed and proposed as therapeutic sites in the treatment of TGCT. The emergence of familial aggregations of TGCT has been described and suggests that hereditary susceptibility exists in the TGCT subset (Greene et al., 2010). In this study, we found “melanoma,” “gastric cancer,” and “breast cancer” pathways were also enriched in KEGG. This finding suggests that the molecular mechanisms of TGCT are partly consistent with other malignant tumors, which tend to have hereditary pathogenic factors as well. For instance, the lower expression of autophagy related proteins and the downregulation of autophagy lead to the acceleration of cancer cell proliferation in TGCT and melanoma (Liu et al., 2018). MiR-223-3p exerts oncogenic influence on TGCT by promoting cell proliferation and inhibiting apoptosis via FBXW7, which is regarded as a tumor suppresser. MiR-223-3p and FBXW7 also act as a target in gastric cancer (Liu et al., 2017). Overexpressed PTTG1 could cause aneuploidy and promotes oncogenesis in breast cancer (Watkins et al., 2010). Meanwhile, PTTG1 overexpression makes seminoma stem cells more invasive and aggravates neoplastic angiogenesis (Grande et al., 2019). The identification of targets in TGCT research can refer to mechanism research in other malignant tumors that possess hereditary susceptibility.

The GSEA results revealed that many pathways correlated with metabolism process, such as amnio acid metabolism (alanine, aspartate, and so on), sugar metabolism, and oxidative phosphorylation process, were enriched as risk score increased. This result was in cooperation with the KEGG results. Studies confirmed that metabolism is involved in the pathogenesis of TGCT. N-acetylcystein has negative impacts on bleomycin-induced apoptosis in malignant TGCTs by inhibiting the mitochondrial pathway, which leads to resistance to apoptosis and might aggravate tumor progression (Cort et al., 2012). Four genes related to glucose metabolism (*LDHA, MCT1, PGK1*, and *TIGAR*) exhibited remarkably high expression in TGCTs, and could be suppressed by microRNA-199a-3p, which might affect aerobic glycolysis and tumorigenesis, via binding to SP1 (Liu et al., 2016). Alterations in the expression level of proteins related to the Warburg effect in adult patients with TGCTs, such as GLUT1 and CD44, suggest the metabolic transformation of malignant cells toward a hyperglycolytic and acid-resistant phenotype in TGCTs, which have a worse outcome (Bonatelli et al., 2019). Researchers found that compared with embryonal carcinoma, the citric acid cycle/mitochondrial oxidative phosphorylation and sphingolipid biosynthesis in TGCT are decreased whereas arachidonic acid metabolism and long-chain fatty acid abundance are increased (Batool et al., 2019). In a word, metabolic reprogramming is common in TGCTs, and cancer therapy that targets metabolic process has brilliant prospects in the future.

P53 pathway, as a classic tumor-associated pathway, was enriched in the high-risk groups. The hyperactivation of p53 effectively enhances the susceptibility of TGCTs to chemotherapy (Kerley-Hamilton et al., 2005). TGCTs showed hypersensitivity to cisplatin because of the apoptosis induction effect of p53 (Gutekunst et al., 2011). The results of these studies imply that p53 activation has a protective effect and influence the effect of cancer treatment.

Some of the six prognostic RBPs are involved in cancer pathogenesis. *DIS3L2*, a conserved exoribonuclease, that plays a part in the degradation of cytoplasmic RNAs, is associated with several cancers. *DIS3L2* promotes hepatocellular carcinoma progression via the regulation of hnRNP U-mediated alterative splicing (Xing et al., 2019). Partial or complete *DIS3L2* deficiency might cause an increased incidence of sporadic Wilms’ tumor (Astuti et al., 2012). *IFIH1*, which acts as a cytosolic receptor, is essential for defense against viral infection by sensing double-stranded RNA (Fischer et al., 2020). *IFIH1* is overexpressed in several topotecan-resistant ovarian cancer cell lines; thus, the gene might participate in the drug resistance mechanism of the tumor (Klejewski et al., 2017). *IGHMBP2* encodes a helicase related to DNA replication and repair (Shen et al., 2006). *IGHMBP2* promotes cell migration and invasion in esophageal squamous carcinoma by downregulating E-cadherin (Chunli et al., 2015). *NPM2* is an oocyte-specific nuclear protein indispensable for nuclear and nucleolar organization. It also participates in early embryonic development processes (Lingenfelter et al., 2011). *NPM2* is hypermethylated and downregulated in melanomas, thus, it might be involved in the early events in the development of malignant melanoma (Fujiwara et al., 2018). *NPM2* is also regarded as a potential immunohistochemical marker via making a distinction between melanoma and benign 5 melanocytic lesions. In a word, these RBPs mentioned are correlated with the development of malignant tumors. *POLR2J3* and *TRMT61A* are rarely found in malignancy before. The chosen RBPs might play a role in the pathogenesis and progression of TGCT, which requires further experiments in the future.

There are several potential reasons why the RBP-based model identified here may be superior to existing prognostic factors in determining the DFS risk for TGCT patients. First of all, the model was based on direct analysis of RBPs, which provides comprehensive and more precise information about the cellular events compared with either clinical parameters or hematological indexes. Second, the data is based on RNA sequencing, which is now a relatively affordable method to obtain an accurate picture of the abundance and activation state of RBPs. Third, the integration of RBPs with other prognostic factors in the nomogram model increased its reliability. Finally, compared with the widely used IGCCCG system, the prognostic predictive model described here contains more valuable information and is more user friendly, suggesting that it may have utility in clinical practice in the future. Based on this risk score, we can predict the DFS of TGCT patients and selected more aggressive therapy for patients with high-risk.

Limitations also exist in our research. First, the predictive efficiency of risk score is unsatisfactory for patients with non-seminoma. Furthermore, we found that this RBP-related risk score applied to seminoma germ cell tumors and did not apply to patients with non-seminoma germ cell tumors. Besides, further study is needed for the specific mechanism of these six RBPs.

## Conclusion

Our results demonstrated that risk score based on six RBPs is associated with poor prognosis in patients with TGCT and can accurately predict prognosis. Risk score along with the serum markers of TGCT could serve as an independent risk factor of TGCT with clinical application in the future.

## Data Availability Statement

Publicly available datasets were analyzed in this study. This data can be found here: The Cancer Genome Atlas (https://portal.gdc.cancer.gov/); the NCBI Gene Expression Omnibus (GSE3218 and GSE10783).

## Author Contributions

NS and XM: protocol/project development. LY and CJ: data collection or management. JL and XZ: data analysis. LY and RC: manuscript writing/editing. All authors read and approved the final manuscript.

## Conflict of Interest

The authors declare that the research was conducted in the absence of any commercial or financial relationships that could be construed as a potential conflict of interest.

## Footnotes

1 http://cancergenome.nih.gov/

2 https://www.gtexportal.org/home/datasets

3 http://string-db.org/

4 https://cytoscape.org/

5 https://www.ncbi.nlm.nih.gov/geo/

6 http://gim.unmc.edu/dxtests/roc3.htm

## Supplementary Material

Supplementary Figure 1 The time-dependent ROC curves for six genes-based risk score, TRMT61A, POLR2J, DIS3L2, IFIH1, IGHMBP2, and NPM2 combining with 1- **(A)**, 3- **(B)**, 5- **(C)**, and 10- **(D)** year DFS in TCGA TGCT cohort, respectively. DFS, disease-free survival. ROC, Receiver operating characteristic curve. TCGA, The Cancer Genome Atlas. TCGA, The Cancer Genome Atlas. DFS, disease-free survival. ROC, Receiver operating characteristic curve. TGCT, Testicular germ cell tumors.

## References

[B1] Astuti D. Morris M. R. Cooper W. N. Staals R. H. Wake N. C. Fews G. A. (2012). Germline mutations in DIS3L2 cause the Perlman syndrome of overgrowth and Wilms tumor susceptibility. *Nat. Genet.* 44 277–284. 10.1038/ng.1071 22306653

[B2] Barlow L. J. Badalato G. M. McKiernan J. M. (2010). Serum tumor markers in the evaluation of male germ cell tumors. *Nat. Rev. Urol.* 7 610–617. 10.1038/nrurol.2010.166 21068762

[B3] Batool A. Chen S. R. Liu Y. X. (2019). Distinct metabolic features of seminoma and embryonal carcinoma revealed by combined transcriptome and metabolome analyses. *J. Proteome Res.* 18 1819–1826. 10.1021/acs.jproteome.9b00007 30835130

[B4] Bonatelli M. Silva E. C. A. Cárcano F. M. Zaia M. G. Lopes L. F. Scapulatempo-Neto C. (2019). The warburg effect is associated with tumor aggressiveness in testicular germ cell tumors. *Front. Endocrinol.* 10:417. 10.3389/fendo.2019.00417 31316469 PMC6610306

[B5] Cheng C. J. Wu Y. C. Shu J. A. Ling T. Y. Kuo H. C. Wu J. Y. (2007). Aberrant expression and distribution of the OCT-4 transcription factor in seminomas. *J. Biomed. sci.* 14 797–807. 10.1007/s11373-007-9198-7 17682839

[B6] Cheng L. Albers P. Berney D. M. Feldman D. Daugaard G. Gilligan T. (2018). Testicular cancer. *Nat. Rev. Dis. Primers* 4:29. 10.1038/s41572-018-0029-0 30291251

[B7] Chovanec M. Cierna Z. Miskovska V. Machalekova K. Kalavska K. Rejlekova K. (2018). βcatenin is a marker of poor clinical characteristics and suppressed immune infiltration in testicular germ cell tumors. *BMC Cancer* 18:1062. 10.1186/s12885-018-4929-x 30390643 PMC6215644

[B8] Chunli W. Jiajie H. Lifei W. Beiqing P. Xin X. Yan C. (2015). [IGHMBP2 overexpression promotes cell migration and invasion in esophageal squamous carcinoma]. *Yi chuan* 37 360–366. 25881701 10.16288/j.yczz.14-371

[B9] Cort A. Timur M. Dursun E. Kucuksayan E. Aslan M. Ozben T. (2012). Effects of N-acetylcystein on bleomycin-induced apoptosis in malignant testicular germ cell tumors. *J. Physiol. Biochem.* 68 555–562. 10.1007/s13105-012-0173-z 22562160

[B10] Diamantopoulos N. Kortsaris A. (2010). Testicular germ cell tumors. *J. BUON* 15 421–434. 20941807

[B11] Dixon F. J. Moore R. A. (1953). Testicular tumors; a clinicopathological study. *Cancer* 6 427–454. 13042768 10.1002/1097-0142(195305)6:3<427::aid-cncr2820060302>3.0.co;2-u

[B12] Einhorn L. H. (2002). Chemotherapeutic and surgical strategies for germ cell tumors. *Chest Surg. Clin. N. Am.* 12 695–706. 10.1016/s1052-3359(02)00029-7 12471872

[B13] Fischer H. Tschachler E. Eckhart L. (2020). Pangolins lack IFIH1/MDA5, a cytoplasmic RNA sensor that initiates innate immune defense upon coronavirus infection. *Front. Immunol.* 11:939. 10.3389/fimmu.2020.00939 32574256 PMC7225364

[B14] Fujiwara S. Nagai H. Jimbo H. Jimbo N. Tanaka T. Inoie M. (2018). Gene expression and methylation analysis in melanomas and melanocytes from the same patient: loss of NPM2 expression is a potential immunohistochemical marker for melanoma. *Front. Oncol.* 8:675. 10.3389/fonc.2018.00675 30719424 PMC6348333

[B15] Gerstberger S. Hafner M. Tuschl T. (2014). A census of human RNA-binding proteins. *Nat. Rev. Genet.* 15 829–845. 10.1038/nrg3813 25365966 PMC11148870

[B16] Glisovic T. Bachorik J. L. Yong J. Dreyfuss G. (2008). RNA-binding proteins and post-transcriptional gene regulation. *FEBS Lett.* 582 1977–1986. 10.1016/j.febslet.2008.03.004 18342629 PMC2858862

[B17] Grande G. Milardi D. Martini M. Cenci T. Gulino G. Mancini F. (2019). Protein expression of PTTG-1, OCT-4, and KLF-4 in seminoma: a pilot study. *Front. Endocrinol.* 10:619. 10.3389/fendo.2019.00619 31572301 PMC6749154

[B18] Greene M. H. Kratz C. P. Mai P. L. Mueller C. Peters J. A. Bratslavsky G. (2010). Familial testicular germ cell tumors in adults: 2010 summary of genetic risk factors and clinical phenotype. *Endocr. Relat. Cancer* 17 R109–R121. 20228134 10.1677/ERC-09-0254 PMC3101798

[B19] Guido C. Panza S. Santoro M. Avena P. Panno M. L. Perrotta I. (2012). Estrogen receptor beta (ERβ) produces autophagy and necroptosis in human seminoma cell line through the binding of the Sp1 on the phosphatase and tensin homolog deleted from chromosome 10 (PTEN) promoter gene. *Cell Cycle (Georgetown Tex)* 11 2911–2921. 10.4161/cc.21336 22810004

[B20] Gutekunst M. Oren M. Weilbacher A. Dengler M. A. Markwardt C. Thomale J. (2011). p53 hypersensitivity is the predominant mechanism of the unique responsiveness of testicular germ cell tumor (TGCT) cells to cisplatin. *PLoS One* 6:e19198. 10.1371/journal.pone.0019198 21532991 PMC3080918

[B21] Hayes-Lattin B. Nichols C. R. (2009). Testicular cancer: a prototypic tumor of young adults. *Semin. Oncol.* 36 432–438. 10.1053/j.seminoncol.2009.07.006 19835738 PMC2796329

[B22] Hong L. K. Chen Y. Smith C. C. Montgomery S. A. Vincent B. G. Dotti G. (2018). CD30-redirected chimeric antigen receptor T cells target CD30(+) and CD30(-) embryonal carcinoma via antigen-dependent and Fas/FasL interactions. *Cancer Immunol. Res.* 6 1274–1287. 10.1158/2326-6066.cir-18-0065 30087115 PMC7590161

[B23] Kerley-Hamilton J. S. Pike A. M. Li N. DiRenzo J. Spinella M. J. (2005). A p53-dominant transcriptional response to cisplatin in testicular germ cell tumor-derived human embryonal carcinoma. *Oncogene* 24 6090–6100. 10.1038/sj.onc.1208755 15940259

[B24] Klejewski A. Świerczewska M. Zaorska K. Brązert M. Nowicki M. Zabel M. (2017). New and old genes associated with topotecan resistance development in ovarian cancer cell lines. *Anticancer Res.* 37 1625–1636. 10.21873/anticanres.11493 28373423

[B25] Lakomý R. Poprach A. Nēmecek R. Vyskocil J. Ondrová B. Kocák I. (2009). [Adjuvant treatment for stage I of testicular germ cell tumours]. *Klin. Onkol.* 22 22–26. 19534436

[B26] Li H. Liang Z. Yang J. Wang D. Wang H. Zhu M. (2019). DAZL is a master translational regulator of murine spermatogenesis. *Natl. Sci. Rev.* 6 455–468. 10.1093/nsr/nwy163 31355046 PMC6660020

[B27] Li L. J. Zhang F. B. Liu S. Y. Tian Y. H. Le F. Lou H. Y. (2014). Decreased expression of SAM68 in human testes with spermatogenic defects. *Fertil. Steril.* 102 61–67.e3. 24794312 10.1016/j.fertnstert.2014.03.036

[B28] Lingenfelter B. M. Tripurani S. K. Tejomurtula J. Smith G. W. Yao J. (2011). Molecular cloning and expression of bovine nucleoplasmin 2 (NPM2): a maternal effect gene regulated by miR-181a. *Reprod. Biol. Endocrinol.* 9:40. 10.1186/1477-7827-9-40 21447182 PMC3072940

[B29] Liu H. He Z. Bode P. Moch H. Simon H. U. (2018). Downregulation of autophagy-related proteins 1, 5, and 16 in testicular germ cell tumors parallels lowered LC3B and elevated p62 levels, suggesting reduced basal autophagy. *Front. Oncol.* 8:366. 10.3389/fonc.2018.00366 30245976 PMC6137693

[B30] Liu J. Shi H. Li X. Chen G. Larsson C. Lui W. O. (2017). miR-223-3p regulates cell growth and apoptosis via FBXW7 suggesting an oncogenic role in human testicular germ cell tumors. *Int. J. Oncol.* 50 356–364. 10.3892/ijo.2016.3807 28000896 PMC5238776

[B31] Liu X. Duan H. Zhou S. Liu Z. Wu D. Zhao T. (2016). microRNA-199a-3p functions as tumor suppressor by regulating glucose metabolism in testicular germ cell tumors. *Mol. Med. Rep.* 14 2311–2320. 10.3892/mmr.2016.5472 27432288

[B32] Lü M. Tian H. Cao Y. X. He X. Chen L. Song X. (2015). Downregulation of miR-320a/383-sponge-like long non-coding RNA NLC1-C (narcolepsy candidate-region 1 genes) is associated with male infertility and promotes testicular embryonal carcinoma cell proliferation. *Cell Death Dis.* 6:e1960. 10.1038/cddis.2015.267 26539909 PMC4670917

[B33] Marshall C. Enzerra M. Rahnemai-Azar A. A. Ramaiya N. H. (2019). Serum tumor markers and testicular germ cell tumors: a primer for radiologists. *Abdom. Radiol. (N. Y.)* 44 1083–1090. 10.1007/s00261-018-1846-z 30539249

[B34] Martinelli C. Lengert A. V. H. Cárcano F. M. Silva E. C. A. Brait M. Lopes L. F. (2017). MGMT and CALCA promoter methylation are associated with poor prognosis in testicular germ cell tumor patients. *Oncotarget* 8 50608–50617. 10.18632/oncotarget.11167 28881587 PMC5584175

[B35] Nettersheim D. Biermann K. Gillis A. J. Steger K. Looijenga L. H. Schorle H. (2011). NANOG promoter methylation and expression correlation during normal and malignant human germ cell development. *Epigenetics* 6 114–122. 10.4161/epi.6.1.13433 20930529 PMC3052918

[B36] Oechsle K. Honecker F. Cheng T. Mayer F. Czaykowski P. Winquist E. (2011). Preclinical and clinical activity of sunitinib in patients with cisplatin-refractory or multiply relapsed germ cell tumors: a canadian urologic oncology group/german testicular cancer study group cooperative study. *Ann. Oncol.* 22 2654–2660. 10.1093/annonc/mdr026 21415240

[B37] Pereira B. Billaud M. Almeida R. (2017). RNA-binding proteins in cancer: old players and new actors. *Trends Cancer* 3 506–528. 10.1016/j.trecan.2017.05.003 28718405

[B38] Pierce G. B. Abell M. R. (1970). Embryonal carcinoma of the testis. *Pathol. Annu.* 5 27–60. 4940001

[B39] Shen J. Terry M. B. Gammon M. D. Gaudet M. M. Teitelbaum S. L. Eng S. M. (2006). IGHMBP2 Thr671Ala polymorphism might be a modifier for the effects of cigarette smoking and PAH-DNA adducts to breast cancer risk. *Breast Cancer Res. Treat.* 99 1–7. 10.1007/s10549-006-9174-3 16752224

[B40] Silván U. Díez-Torre A. Moreno P. Arluzea J. Andrade R. Silió M. (2013). The spermatogonial stem cell niche in testicular germ cell tumors. *Int. J. Dev. Biol.* 57 185–195. 10.1387/ijdb.130068ja 23784829

[B41] Subramanian A. Tamayo P. Mootha V. K. Mukherjee S. Ebert B. L. Gillette M. A. (2005). Gene set enrichment analysis: a knowledge-based approach for interpreting genome-wide expression profiles. *Proc. Natl. Acad. Sci. U.S.A.* 102 15545–15550. 10.1073/pnas.0506580102 16199517 PMC1239896

[B42] Terbuch A. Posch F. Bauernhofer T. Pichler M. Peinsith H. Szkandera J. (2019). Age as a predictor of treatment outcome in metastatic testicular germ cell tumors. *Anticancer Res.* 39 5589–5596. 10.21873/anticanres.13753 31570454

[B43] Togawa K. Le Cornet C. Feychting M. Tynes T. Pukkala E. Hansen J. (2016). Parental occupational exposure to heavy metals and welding fumes and risk of testicular germ cell tumors in offspring: a registry-based case-control study. *Cancer Epidemiol. Biomarkers Prev.* 25 1426–1434. 10.1158/1055-9965.epi-16-0328 27439405

[B44] Tsili A. C. Sofikitis N. Stiliara E. Argyropoulou M. I. (2019). MRI of testicular malignancies. *Abdom. Radiol. (N. Y.)* 44 1070–1082. 10.1007/s00261-018-1816-5 30386879

[B45] Watkins R. J. Read M. L. Smith V. E. Sharma N. Reynolds G. M. Buckley L. (2010). Pituitary tumor transforming gene binding factor: a new gene in breast cancer. *Cancer Res.* 70 3739–3749. 10.1158/0008-5472.can-09-3531 20406982 PMC2875163

[B46] Xing S. Li Z. Ma W. He X. Shen S. Wei H. (2019). DIS3L2 promotes progression of hepatocellular carcinoma via hnRNP U-mediated alternative splicing. *Cancer Res.* 79 4923–4936. 10.1158/0008-5472.can-19-0376 31331910

[B47] Yu J. Navickas A. Asgharian H. Culbertson B. Fish L. Garcia K. (2020). RBMS1 suppresses colon cancer metastasis through targeted stabilization of its mRNA regulon. *Cancer Discov.* 10 1410–1423. 10.1158/2159-8290.cd-19-1375 32513775 PMC7483797

[B48] Zhang T. Ji L. Liu B. Guan W. Liu Q. Gao Y. (2018). Testicular germ cell tumors: a clinicopathological and immunohistochemical analysis of 145 cases. *Int. J. Clin. Exp. Pathol.* 11 4622–4629. 31949861 PMC6962990

[B49] Zhao J. Zhang Y. Liu X. S. Zhu F. M. Xie F. Jiang C. Y. (2020). RNA-binding protein Musashi2 stabilizing androgen receptor drives prostate cancer progression. *Cancer Sci.* 111 369–382. 10.1111/cas.14280 31833612 PMC7004550

