# Supplementary figures and images for: RNA-Binding Proteins Play an Important Role in the Prognosis of Patients With Testicular Germ Cell Tumor
Source: Front Genet. 2021 Mar 11;12:610291. doi: 10.3389/fgene.2021.610291 (PMC7990889; doi:10.3389/fgene.2021.610291)

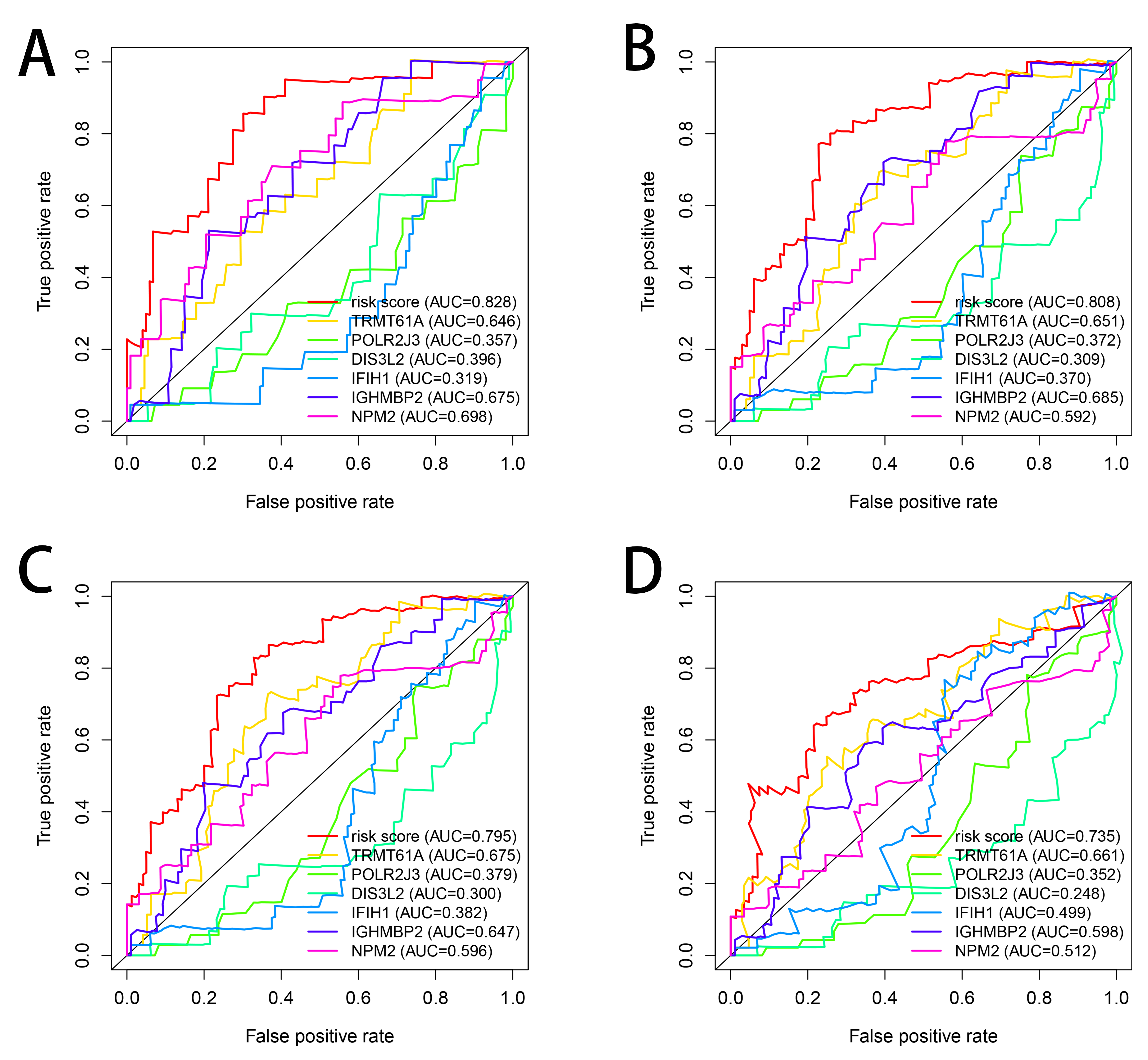

Supplement: Supplementary Figure 1 — The time-dependent ROC curves for six genes-based risk score, TRMT61A, POLR2J, DIS3L2, IFIH1, IGHMBP2, and NPM2 combining with 1- (A), 3- (B), 5- (C), and 10- (D) year DFS in TCGA TGCT cohort, respectively. DFS, disease-free survival. ROC, Receiver operating characteristic curve. TCGA, The Cancer Genome Atlas. TCGA, The Cancer Genome Atlas. DFS, disease-free survival. ROC, Receiver operating characteristic curve. TGCT, Testicular germ cell tumors. [file Image_1.TIF]
